# Unusual presentation of angiomyomatous hamartoma in an eight-month-old infant: case report and literature review

**DOI:** 10.1186/1471-2431-12-172

**Published:** 2012-11-06

**Authors:** Vincenzo Davide Catania, Carlo Manzoni, Mariangela Novello, Libero Lauriola, Antonella Coli

**Affiliations:** 1 Department of Pediatric Surgery, Catholic University of Sacred Heart, Largo A.Gemelli 8, Rome, 00168, Italy; 2 Department of Anatomic Pathology, Catholic University of Sacred Heart, Largo A. Gemelli 8, Rome, 00168, Italy

**Keywords:** Angiomyomatous hamartoma, Pediatric neck mass, Ultrasonography, Lymph node

## Abstract

**Background:**

Evaluation of palpable neck masses may be a diagnostic problem in pediatric patients, with differential diagnosis including congenital, inflammatory, tumoral and traumatic lesions. Ultrasonography is usually a satisfactory method to make a correct pre-operative evaluation of neck masses, although diagnosis is often challenging for the surgeon and the radiologist and sometimes only possible after a histopathological examination of the resected lesion.

**Case presentation:**

We report an 8-month-old patient with a cervical, anterior midline mass. Ultrasonographic images showed features suggesting a partly cystic lesion, with a preoperative suspect of thyroglossal duct cyst. Histological examination, performed after surgical removal of the mass, led to a diagnosis of lymph node angiomyomatous hamartoma (AH).

**Conclusions:**

AH, a rarely occurring benign lymph node lesion, has been reported in the neck lateral region only twice. This case, presenting as a palpable neck midline mass, is the first reported case occurring in infancy. Although rare, AH should be included in the differential diagnosis of head and neck masses.

## Background

Palpable neck masses are a common clinical concern in pediatric patients, and differential diagnosis includes a wide range of pathologies, such as congenital, inflammatory, tumoral and traumatic lesions. Malformative neck masses in childhood are branchial cleft cysts, ectopic thymus, teratomas, cystic lymphangioma, aberrant thyroid tissue and others [1-6]. Thyroglossal duct cyst is a congenital anomaly related to persistence of thyroglossal duct, presenting as a midline cyst, near the hyoid bone with variable sonographic appearances [7,8]. AH is a rare, benign lymph node pathology consisting of a fibrous transformation of the hilar region, also with presence of vessels and smooth muscle cell proliferation. Of 28 cases hitherto reported, only two were located in the lateral neck region, both in adult patients, and the lesion has been more frequently found in the inguinal and femoral region [9-11].

Ultrasonography (US) is an accurate, cost-effective, non-invasive preoperative analysis, usually sufficient to make correct preoperative diagnoses, even if the accuracy of this imaging method may have limitations, because of the variability of the sonographic appearances [12,13]. Computed tomography and magnetic resonance imaging play a supplementary diagnostic role. Obviously, surgical excision with histological examination allows a definite diagnosis.

The authors herein describe an unusual case of AH presenting as a palpable mass in the anterior midline neck region of an infant. US preoperative evaluation of the lesion appeared compatible with a partly cystic mass, and, due to the peculiar location, a thyroglossal duct cyst was suspected or, secondly, a dermoid cyst, a small teratoma or an enlarged reactive lymph node.

## Case presentation

**Figure 1:**
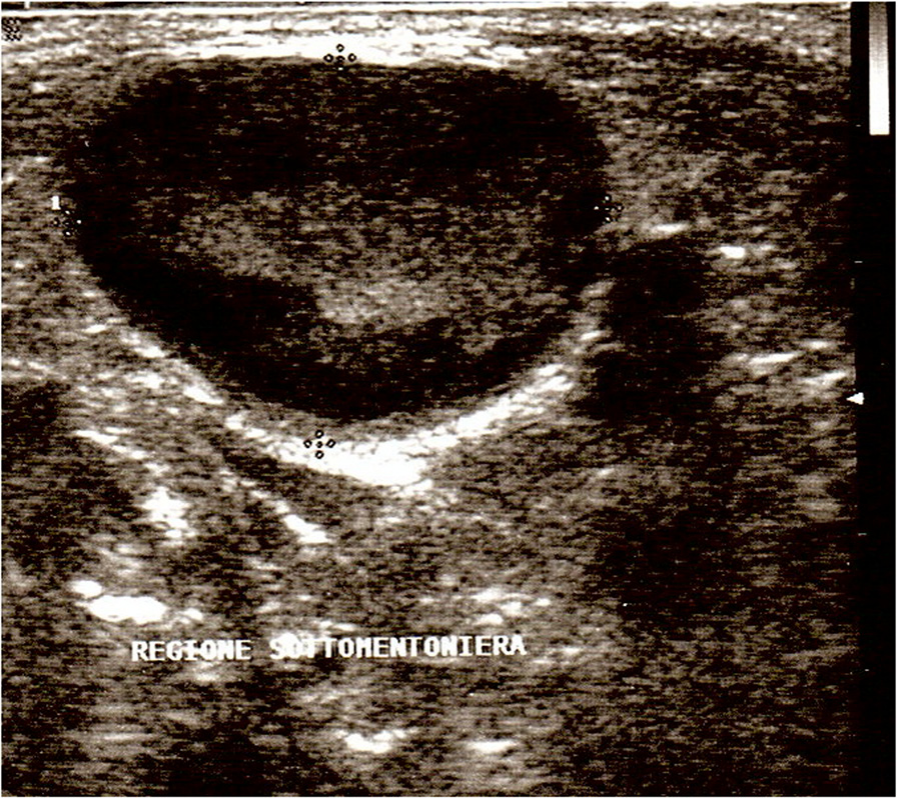
**Ultrasonographic findings in an 8-month-old girl with a midline neck lesion.** Sonogram shows a well-defined oval mass characterized by a hypoechoic peripheral layer and heterogeneous internal echoes.

An 8-month-old Philippine female baby without other health problems presented with a palpable neck mass of 4 months’ duration. The patient did not suffer from dysphagia nor dysphonia. The past medical history was unremarkable. On physical examination, a midline anterior neck mass, tender and mobile when swallowing, contiguous to the hyoid bone was observed. No enlarged lymph nodes were detected in the lateral neck region. Laboratory values were in normal range. At US, a lesion largely hypoechoic measuring 20 x 14 mm in diameter, with internal heterogeneous echoes, with a well defined periphery (Figure 1) was detected and the thyroid gland did not show abnormalities. A thyroglossal duct cyst was suspected and the patient underwent surgical excision. Intraoperatively we couldn’t differentiate if the lesion was a solid mass or a cystic one and its malignant potential, if any, so that the Sistrunk procedure was chosen for safety’s sake. A careful dissection of the lesion was performed, and the central portion of the hyoid bone was resected. In the postoperative course, the patient suffered from dyspnea and dysphonia, with relief of symptoms on the third post-operative day. At follow-up, 8 months after surgery, the patient was well, without evidence of disease.

**Figure 2:**
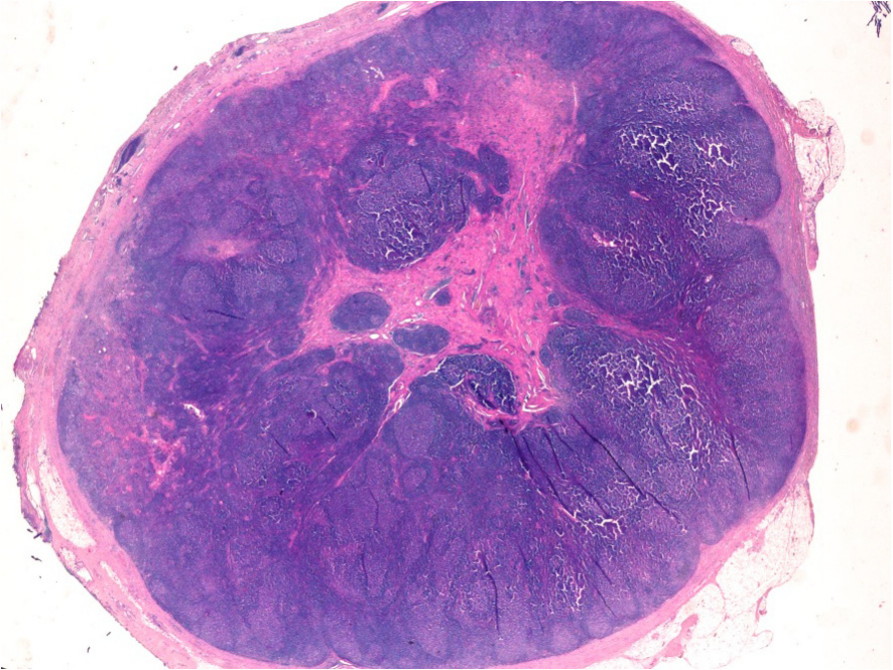
**Low-magnification image of lymph node affected by angiomyomatous hamartoma.** The hilar region is replaced by a dense eosinophilic tissue, leaving a residual rime of cortical lymphoid tissue. Note the striking contrast between the central area and the normal peripheral tissue. Original magnification, x 5; hematoxylin-eosin stain.

**Figure 3:**
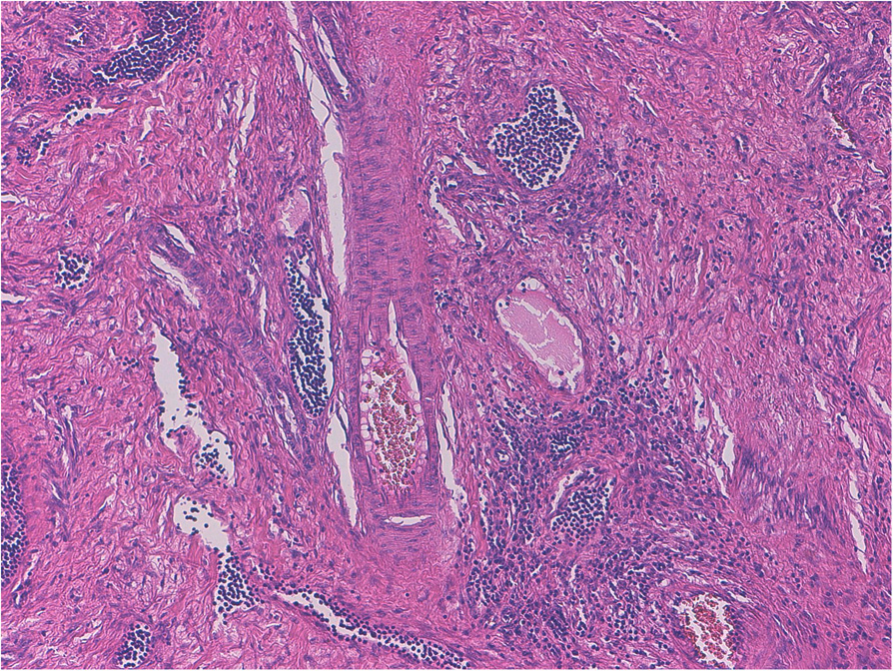
**At higher magnification, the inner area of the lymph node shows irregular vessels and smooth muscle cells haphazardly interspersed throughout a collagenous stroma.** Original magnification, x 100; hematoxylin-eosin stain.

On gross examination, the excised mass measured 20 mm in largest diameter; a central fibrous areas was detected, without evidence of cyst. The tissue was fixed in 10% buffered formalin, routinely processed and paraffin embedded. Histological examination revealed a preserved lymph node structure at the periphery, with reactive follicles and dilated sinuses, whereas, in the region of the hilum, the nodal parenchyma was completely replaced by a fibrous tissue (Figure 2) containing numerous irregular vessels and spindle cell bundles (Figure 3). On immunohistochemical analysis, the spindle cells appeared as smooth muscle fibers, as confirmed by their positivity for HHF35 and desmin. Consecutive sections did not document presence of a cystic lesion. All the aforementioned findings were consistent with a pathological diagnosis of AH of the lymph node.

## Conclusions

Neck lesions more commonly encountered in pediatric population consist of congenital, inflammatory or neoplastic diseases [3,5]. In addition to branchial cleft cysts, located in the lateral neck, malformative midline lesions prevalently belong to thyroglossal duct sinus and cyst, and present with a variable US appearance, ranging from a typical anechoic cyst to a pseudosolid lesion [7,8].

AH is a rare, benign form of lymph node hilar modification characterized by parenchymal replacement by fibrous tissue containing vessels and smooth-muscle proliferation. It prevalently involves inguinal lymph nodes, appearently starting in the hilum and extending towards the cortex. This entity was first described by Chan et al. in 1992, and, so far, 28 cases have been reported in the literature [9-11,14-23]. Table 1 shows age, gender and location of the lesion in 28 patients.

**Table 1:** Cases of angiomyomatous hamartoma reported in literature

**Case n.**	**Author, year**^**(ref)**^	**Age (y)**	**Sex**	**Site**
1-12	Chan et al., 1992 [9]	80	M	L inguinal
		24	M	L inguinal
		10	F	L inguinal
		19	M	L inguinal
		19	M	R inguinal
		50	F	L inguinal
		31	M	R inguinal
		56	M	R inguinal
		60	M	L inguinal
		56	M	L inguinal
		52	M	R inguinal
		44	M	R femoral
13	Allen et al., 1993 [14]	67	F	R femoral
14	Laeng et al., 1996 [10]	17	F	L latero-cervical
15	Magro et al., 1997 [15]	68	M	L inguinal
16	Sakurai et al., 2000 [16]	51	M	R inguinal
17	Dargent et al., 2004 [17]	29	M	R inguinal
18	Piedimonte et al., 2006 [18]	34	F	L inguinal
19	Sullu et al., 2006 [19]	33	F	R inguinal
20	Mauro et al., 2008 [20]	41	M	L popliteal
21	Barzilai et al., 2009 [11]	51	F	L latero-cervical
22-26	Bourgeois et al., 2009 [21]	8	M	L inguinal
		15	F	L inguinal
		30	M	R inguinal
		50	M	L inguinal
		57	M	R inguinal
27	Ram et al., 2009 [22]	82	M	L inguinal
28	Prusac et al., 2011 [23]	14	M	R popliteal
29	Present case	8 months	F	Midline cervical

In our infant patient, AH occurred in a midline neck lymph node. To our knowledge, only two cases, both in adults, have been documented in the lateral neck region. The ultrasound image of the lump yielded a suspected cystic lesion, for the variable tissue density and for the presence of an internal fibro-vascular area contrasting with the intact lymph node periphery and providing the hypothesis of thyroglossal duct cyst. The histological examination, performed after excision, allowed the correct diagnosis.

The pathogenesis of this lesion is unclear. A possible explanation is that AH represents a vascular and smooth muscle proliferative response to chronic impairment of nodal lymphatic flow or to previous nodal inflammation [9,16].

Notwithstanding the rarity, AH should be included in the differential diagnosis of neck masses. The treatment of choice for this benign disease is surgical excision. The Sistrunk procedure, performed in our case, is a safe and successful technique with low complications.

### Consent

Written informed consent was obtained from the parents for publication of this case report and any accompanying images.

## Competing interests

The authors declare that they have no competing interests.

## Authors’ contributions

VDC and AC conceived the study and drafted the manuscript. VDC participated in surgery. LL has been involved in drafting and critical revision of the manuscript. CM did surgery and revised the manuscript. MN was involved in preparation of figures and manuscript revision. All authors read and approved the final manuscript.

## Pre-publication history

The pre-publication history for this paper can be accessed here:

http://www.biomedcentral.com/1471-2431/12/172/prepub
